# Effect of salicylic acid on morphological traits and the expression of key rubber biosynthesis genes in *Parthenium argentatum* A. Gray

**DOI:** 10.1371/journal.pone.0358720

**Published:** 2026-10-01

**Authors:** Fatemeh Mohammadi, Mohammad Reza Naghavi, Mohtaram Mahmoudieh, Meisam Zargar

**Affiliations:** 1 Division of Biotechnology, Department of Agronomy and Plant Breeding, College of Agricultural and Natural Resources, University of Tehran, Karaj, Iran; 2 Department of Agrobiotechnology, Agrarian Technological Institute, RUDN University, Moscow, Russia; Abdul Wali Khan University Mardan, PAKISTAN

## Abstract

Guayule (*Parthenium argentatum* A. Gray) serves as a critical, drought-tolerant alternative source of natural rubber for medical and industrial applications. While salicylic acid is a fundamental signaling molecule in plant development, stress responses, and secondary metabolism, its specific influence on the gene expression of rubber biosynthesis in guayule remains poorly understood. This study evaluated the effects of exogenous salicylic acid on the morphological traits and regulation of cis-prenyltransferase 3 (*CPT3*), small rubber particle protein (*SRPP*), farnesyl pyrophosphate synthase (*FPPS*), and allene oxide synthase (*AOS*) in guayule seedlings grown *in vitro* condition. Seedlings at the four-leaf stage were treated with varying concentrations of salicylic acid (0, 2.5, 5, and 7.5 µM) for four weeks. Treatment with exogenous salicylic acid induced a biphasic morphological response in *P*. *argentatum* seedlings. Morphological analysis revealed a dose-dependent response: although moderate concentrations (2.5 and 5 µM) showed a trend toward increased shoot and root elongation, these differences were not statistically significant. However, 7.5 µM inhibited elongation but significantly stimulated leaf and shoot proliferation. This dose-dependent shift highlighted the hormetic potential of salicylic acid in modulating plant morphogenesis without affecting seedling viability. In addition, quantitative gene expression analysis showed significant, concentration-dependent upregulation of *CPT3* and *SRPP*, alongside gradual increases in *AOS* expression. However, *FPPS* was significantly induced only at 7.5 µM salicylic acid. These results demonstrate that salicylic acid is a potent modulator of both vegetative growth and the genes expression of rubber biosynthesis pathway. Consequently, these findings provide a molecular foundation for employing hormonal elicitors to upregulate key biosynthetic genes in guayule biotechnology.

## Introduction

Natural rubber is an essential industrial raw material widely used in automotive, medical, and manufacturing industries due to its superior elasticity, resilience, and mechanical properties [1]. Currently, global natural rubber production relies predominantly on *Hevea brasiliensis*, a species that requires humid tropical conditions and is highly vulnerable to diseases such as South American Leaf Blight (SALB), which poses a serious threat to rubber production worldwide [2,3]. These limitations highlight the urgent need to explore alternative and sustainable sources of natural rubber [4,5].

Guayule, (*Parthenium argentatum* A. Gray) is a perennial shrub native to arid and semi-arid regions of the southwestern United States and northern Mexico and has emerged as a promising alternative source of natural rubber [6]. Unlike *H. brasiliensis*, guayule is drought-tolerant, adaptable to calcareous soils, and produces hypoallergenic latex free of allergenic proteins commonly found in Hevea latex [1]. These characteristics make guayule a strategic crop for cultivation in dry and semi-arid regions [7]. Rubber biosynthesis in guayule involves a complex terpenoid regulated by several key genes, including cis-prenyltransferase (*CPT*), small rubber particle protein (*SRPP*), farnesyl pyrophosphate synthase (*FPPS*), and allene oxide synthase (*AOS*) [3,8]. The physiological role of *AOS* remains a subject of debate, with hypotheses suggesting either a direct or indirect involvement in rubber biosynthesis [9] or a primary structural function within the rubber particle [10]. Although the physiological and agronomic aspects of guayule have been extensively studied, information regarding the molecular regulation of rubber biosynthesis, particularly under hormonal treatments, remains limited [3,11]. Stonebloom and Scheller [11] identified *FPPS* as part of a cold-responsive rubber gene set; however, direct evidence for its regulation by hormones, particularly salicylic acid, has remained limited in rubber-producing species.

Salicylic acid (a phenolic compound) is a well-known plant signaling molecule involved in the regulation of growth, development, stress responses, and secondary metabolism [12]. Placido et al., [13] reported that rubber accumulation in *P*. *argentatum* increased in stem bark tissues under cold and wounding stress conditions and was accompanied by elevated endogenous salicylic acid levels and reduction jasmonic acid concentrations. Under drought stress, salicylic acid treatment upregulated isopentenyl diphosphate isomerase (*IPPI*) and mitigated the drought-induced suppression of key terpenoid biosynthetic genes, including geranyl diphosphate synthase (*GPPS*), 3-hydroxy-3-methylglutaryl coenzyme A reductase (*HMGR*), farnesyl pyrophosphate synthase (*FPS*), and β-amyrin synthase (*βAS*). In addition, combined salicylic acid and silicon application significantly enhanced the accumulation of β-carotene, α-tocopherol, and β-amyrin in *Scrophularia striata* [14]. Furthermore, salicylic acid treatment increased the accumulation of terpenoid-derived secondary metabolites in *Silybum marianum* [15].

Research on *H*. *brasiliensis* has shown that ethylene- and jasmonate-mediated signaling modules, together with transcriptional networks and co-expression analyses, play important roles in the hormonal regulation of latex production and rubber biosynthesis [16–18]. However, in *P*. *argentatum*, the direct effects of exogenous salicylic acid on the expression of key genes involved in natural rubber biosynthesis have not been thoroughly investigated. Despite reported stress-related changes in endogenous salicylic acid in *P*. *argentatum* [13], the influence of exogenous treatment on rubber biosynthesis gene expression and seedling morphology remains unclear. This study provides the systematic evaluation of exogenous salicylic acid effects on both the morphological traits and the expression profiles of the *CPT3*, *SRPP*, *FPPS* and *AOS* genes in *P*. *argentatum* grown in vitro. By establishing an association between exogenous salicylic acid application and the transcriptional regulation of these key pathway genes, our findings provide novel insights into hormone-mediated transcriptional regulation of rubber biosynthesis-related genes in guayule.

## Materials and methods

### Plant material, experimental design, and salicylic acid treatments

*P*. *argentatum* seeds (Seed Bank code number: UC100), obtained from the Seed Bank of the Research Institute of Forests and Rangelands, Karaj, Iran, were surface-sterilized as described previously by George et al., [19]. Briefly, the seeds were immersed in 1% (v/v) sodium hypochlorite solution containing 0.01% (v/v) Tween 20 for 5 minutes, followed by three rinses with sterile distilled water [19]. Sterilized seeds were cultured on Murashige and Skoog (MS) basal medium [20] supplemented with 3% (w/v) sucrose and solidified with 0.8% (w/v) agar for germination in a controlled growth room at 22 ± 2 °C under a 16/8 h (light/dark) photoperiod [21].

For salicylic acid treatments, guayule seedlings at the four-leaf stage (approximately 3–4 cm in length) were cultured on full-strength MS medium supplemented with 0 (control seedlings), 2.5, 5, and 7.5 µM salicylic acid. Salicylic acid stock solutions were prepared in 65% ethanol prior to addition to the culture medium. All seedlings were maintained under controlled growth room conditions for four weeks. Each treatment consisted three biological replicates (n = 3), with four seedlings per replicate. A summary of the experimental design is presented in Fig 1.

**Fig 1:**
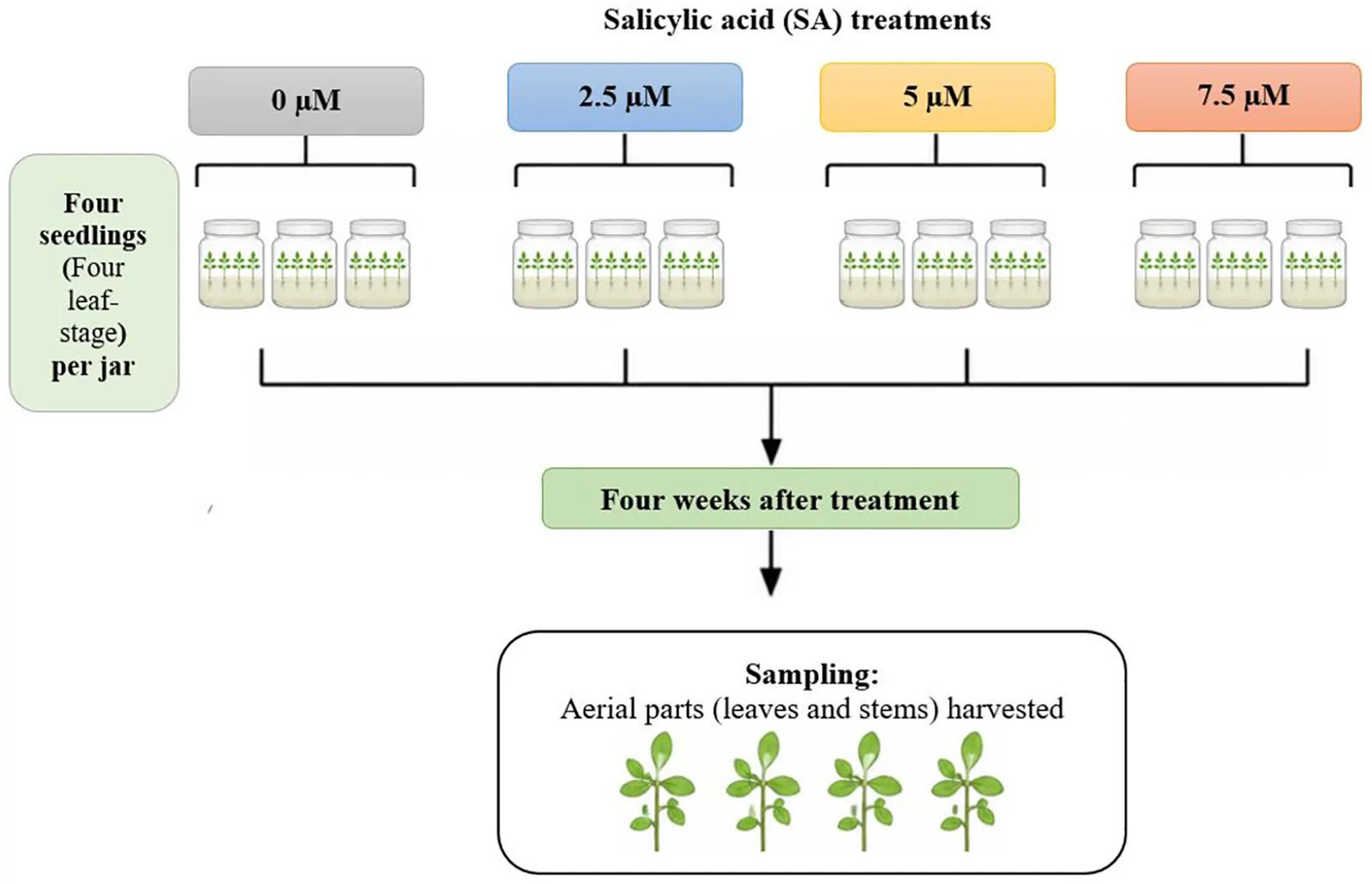
Schematic representation of the experimental design for salicylic acid treatments of *Parthenium argentatum* seedlings. Seedlings grown in MS medium, were treated with different concentrations of salicylic acid (0 as control, 2.5, 5, and 7.5 µM). Each treatment consisted of three biological replicates (n = 3; each jar containing 4 seedlings serving as a separate replicate). Samples were harvested four weeks after treatment for subsequent analysis.

### Morphological traits assessment

After four weeks of salicylic acid treatment, morphological parameters were assessed. The number of shoots and visible roots (including primary and lateral roots) and leaves per seedlings were recorded. Shoot length was measured from the stem base to the apical meristem, and root length was measured from the stem base to the tip of the longest root using a standard ruler (±1 mm precision).

### Quantitative real-time PCR analysis

Total RNA was extracted from approximately 100 mg of tissue, including leaves and stems, collected from three biological replicates (n = 3; each jar containing 4 seedlings serving as a separate replicate). For each biological replicate, tissue from the four seedlings within the same jar was pooled prior to RNA isolation. RNA extraction was performed using Total RNA isolation kit (DenaZist, Asia, Iran) according to the manufacturer’s instructions. The RNA was quantified with a Nanodrop spectrophotometer (Nanodrop ND-1000) and assessed using 1% agarose gel electrophoresis. DNase treatment was then performed using Deoxyribonuclease I (DNase I, SinaClon, Iran) to eliminate any residual genomic DNA contamination. Reverse transcription was carried out using a reverse transcription kit (cDNA Synthesis Kit Parstous, Iran) following the manufacturer’s instructions to convert RNA into complementary DNA (cDNA). Quantitative real-time PCR was performed in three biological replicates (n = 3) for each cDNA sample using a SYBR Green master mix (TQ1100] ExcelTaq™ 2X Q-PCR Master Mix (Parstous, Iran) in a Rotor-Gene 6000 series system (QIAGEN’s real time PCR system) to analyze the expression levels of *CPT3* (cis-prenyltransferase 3), *SRPP* (small rubber particle protein), *AOS* (allene oxide synthase), and *FPPS* (Farnesyl pyrophosphate synthase) genes. The thermal cycling conditions were performed according to the manufacturer’s instructions as follows: an initial denaturation at 95 °C for 15 minutes, followed by 42 cycles of denaturation at 95 °C for 20 seconds, annealing at 59−60 °C (depending on the annealing temperature) for 20 seconds, and extension at 72 °C for 30 seconds. *Actin* gene was used as the internal reference gene to normalize gene expression levels. The following combinations of forward and reverse primers were used: for the quantitative real-time PCR for *CPT3* 5′-GCTTCTTTTTCGGGTCATTTCA-3′/5′-TGCCAAGAATCCGGCTTTAT-3′; for *SRPP* 5′-GTGGCCAACACATTGTACGTAAA-3′/5′-TTCTCAGCTACCGGCTCGTAGT-3′; for *FPPS* 5′-TCAACGATCCTGCCTTCGA-3′/5′-TCCAGGTACGTTGTAGTCAAGCA-3′; for *AOS* 5′-CACGGTATTTCGAGCCAACA-3′/5′-CGTCGAGTAGGACGATTACCTT-3′ [22]; for *Actin* 5′-GTATCCATGAGACCACCTACAAC-3′/5′-GTCAGCAATACCAGGGAACATA-3′.

### Statistical analysis

All experiments were conducted using a completely randomized design (CRD). Morphological traits and gene expression data were subjected to analysis of variance (ANOVA) using SAS software (version 9.4). Mean comparisons were performed using Tukey’s honestly significant difference test (HSD) at a significance level of *p* ≤ 0.05 [23,24]. Relative gene expression levels were calculated using the 2^−ΔΔC^^t^ method **[**25**]**.

### Inclusivity in global research

Additional information regarding the ethical, cultural, and scientific considerations specific to inclusivity in global research is included in the Supporting Information (S1 File).

## Results

### Effect of salicylic acid on morphological traits of *P*. *argentatum*

Exogenous application of salicylic acid affected several morphological traits of *P*. *argentatum* seedlings grown under in vitro conditions, including leaf and shoot numbers, as well as root and shoot lengths (Fig 2). The results showed that root length varied among salicylic acid concentrations. The seedlings treated with 5 µM salicylic acid exhibited the longest roots (4 cm), whereas the shortest roots (1.5 cm) were observed under 7.5 µM treatment (*p* ≤ 0.05). The root lengths in the 2.5 µM salicylic acid treatment (3 cm) did not differ significantly from control group (2 cm). However, shoot length was also significantly influenced by salicylic acid concentration; maximum shoot length was observed at 5 µM, while the 7.5 µM treatment resulted in a significant reduction in shoot length compared to the 5 µM treatment (Fig 2A). Furthermore, salicylic acid treatment significantly affected the number of leaves and shoots (*p* ≤ 0.05), with the highest numbers observed at 7.5 µM salicylic acid relative to the control; however, root number was not significantly altered by any treatment (Fig 2B).

**Fig 2:**
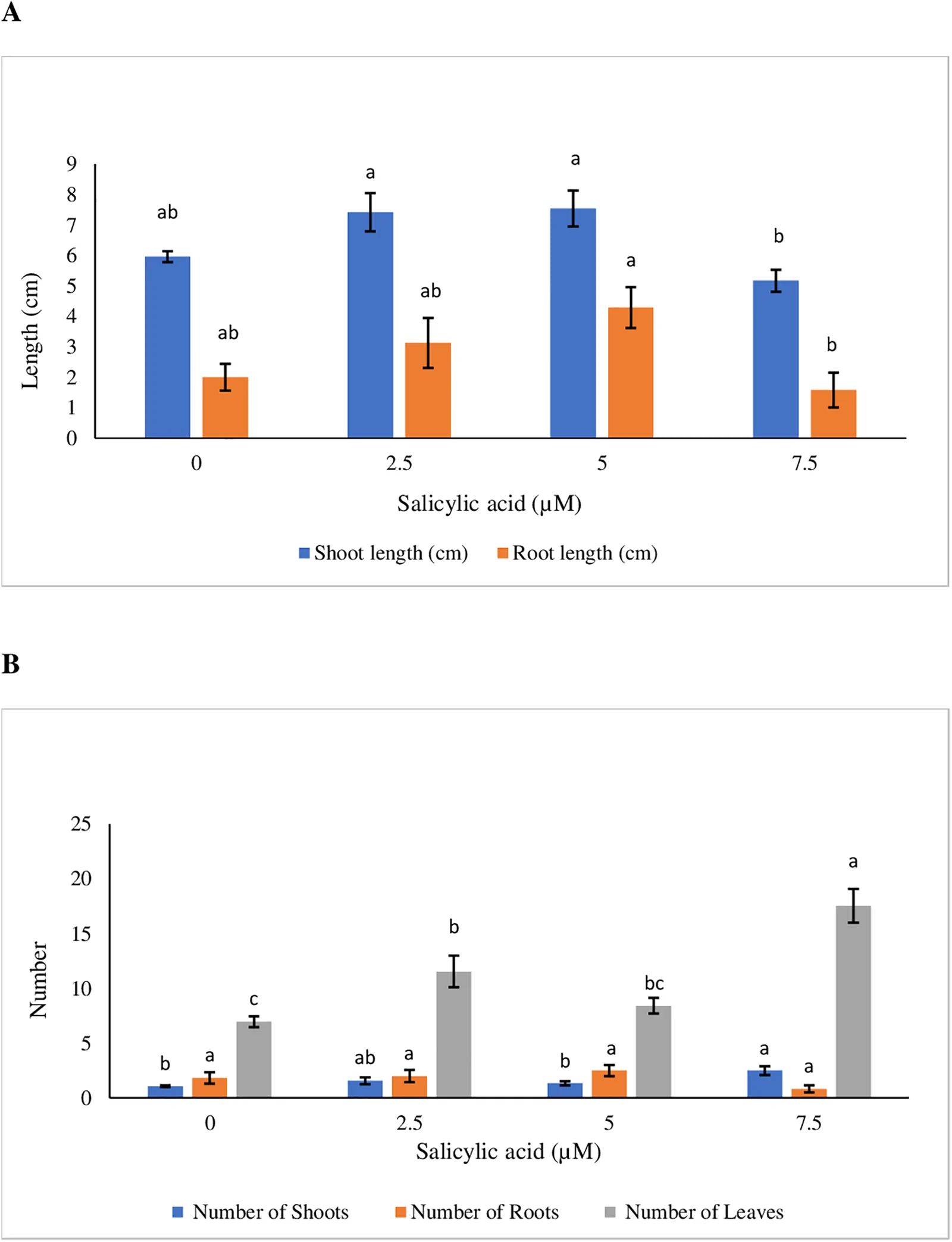
The effect of salicylic acid on morphological traits of *P*. *argentatum.* The impact of different concentrations of salicylic acid on root and shoot length (cm; **A**), number of roots, shoots and leaves **(B)** of *P*. *argentatum* are shown. The values are presented as mean ± standard error (SE) of 3 replications (n = 3; each jar containing 4 seedlings serving as a separate replicate). The values followed by the same letter are not significantly different according to Tukey’s test (*p* ≤ 0.05).

### Impact of salicylic acid on the expression of rubber biosynthesis genes

The results showed that salicylic acid treatments significantly (*p* ≤ 0.05) altered the expression level of key genes involved in rubber biosynthesis in guayule seedlings (Fig 3). Cis*-*prenyltransferase 3 (*CPT3*) gene expression exhibited a significant, dose-dependent induction in response to salicylic acid (Fig 3A). Expression of *CPT3* gene was low in the control seedlings and maximal at 7.5 µM, a level statistically higher than all other treatments (approximately a 20-fold change). The expression of small rubber particle protein (*SRPP*) was markedly enhanced across all salicylic acid concentrations relative to the control group, with the maximal transcript accumulation occurring at 7.5 µM salicylic acid (more than a 5-fold change; Fig 3B). While moderate salicylic acid concentrations also induced significant increases, the magnitude of this upregulation was lower than that observed for *CPT3*. The expression analysis for farnesyl pyrophosphate synthase (*FPPS*) showed that low and moderate salicylic acid concentrations elicited only minor increases in *FPPS* transcript levels (Fig 3C). However, a significant upregulation of *FPPS* (more than a 3-fold change) was specifically observed at the 7.5 µM salicylic acid compared to the control group, indicating that *FPPS* responsiveness is highly dependent on higher salicylic acid dosing. Allene oxide synthase (*AOS*) gene expression was significantly affected by the salicylic acid treatment (p<0.0001; Fig 3D). Consistent with *SRPP* and *FPPS* expressions, a gradual escalation in *AOS* expression was observed with increasing salicylic acid concentration, resulting in peak expression at 7.5 µM (more than a 14-fold change; Fig 3).

**Fig 3:**
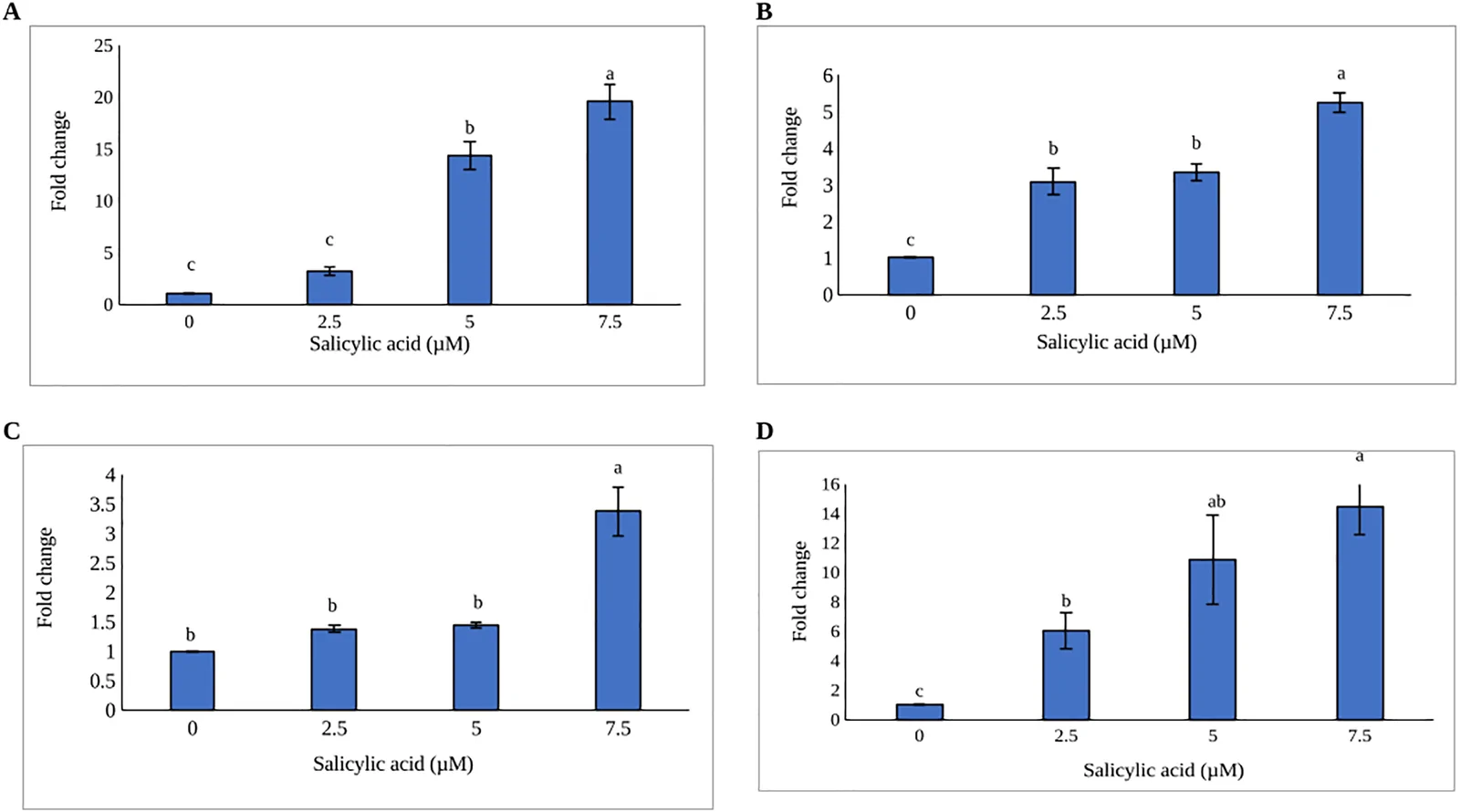
Quantitative real-time PCR analysis for expression of natural rubber biosynthesis genes. The genes analyzed were cis-prenyltransferase 3 (*CPT3*; **A**), small rubber particle protein (*SRPP*; **B**), farnesyl pyrophosphate synthase (*FPPS*; **C**), and allene oxide synthase (*AOS*; **D**). Different concentrations of salicylic acid (0, 2.5, 5, and 7.5 µM) were applied. The values are presented as mean ± standard error (SE) of the average of biological replicates (n = 3). The values followed by different letters are significantly different (*p* ≤ 0.05).

## Discussion

### Modulation of *P*. *argentatum* morphological traits by salicylic acid

In this study, the application of salicylic acid resulted in marked changes in the morphology of guayule seedlings cultured in vitro. These findings highlighted the potential of salicylic acid as a growth‑modulating agent and supported earlier reports on its role in morphogenesis and stress tolerance [26]. Our results showed that 5 µM salicylic acid was the most effective concentration for promoting shoot and root elongation, indicating a stimulatory effect on vegetative growth. This result was consistent with previous reports describing a concentration-dependent role of salicylic acid in plant growth regulation, where moderate concentrations enhanced elongation, while higher concentrations suppressed growth-related processes [27–29]. Exposure to the highest salicylic acid concentration (7.5 µM) resulted in a marked reduction in shoot and root elongation, accompanied by a significant increase in shoot and leaf numbers. Notably, no visible stress symptoms such as chlorosis, necrosis, or wilting were observed, and the seedlings remained viable throughout the experimental period. This biphasic response suggested a hormetic effect, in which lower concentrations promote morphogenesis while higher levels inhibit specific growth parameters. Similar dose-dependent morphological adjustments have been reported in association with defense signaling activation and shifts toward secondary metabolism [27–29]. A study by Ortega‑Macareno and Iglesias‑Andreu [21] in *Vanilla planifolia* reported that salicylic acid increased shoot and root elongation under in vitro conditions. However, in the present study, the highest salicylic acid concentration (7.5 µM) reduced both shoot and root elongation in guayule seedlings. These contrasting responses likely reflect species- and genotype-specific sensitivity to salicylic acid, as its biosynthesis and signaling pathways differ among plant species, limiting broad generalizations [30]. Moreover, extensive evidence indicated that salicylic acid interacted antagonistically or synergistically with other phytohormones, including auxin and abscisic acid, thereby modulating growth–defense trade-offs under varying physiological conditions [31]. Specifically, the reduced shoot and root elongation at 7.5 µM salicylic acid may reflect the context-dependent antagonism between salicylic acid and auxin signaling, as Arif et al., [31] reported that elevated salicylic acid can induce auxin-deficiency-like phenotypes and suppress auxin responses. Nevertheless, the concurrent increase in shoot and leaf proliferation suggests that salicylic acid may differentially regulate cell elongation and organ initiation, potentially through crosstalk with other growth-related hormones [31]. These findings highlight the importance of considering species‑specific hormonal response profiles and suggest that quantifying endogenous auxin levels is essential when evaluating the physiological effects of salicylic acid across diverse plant taxa.

### Salicylic acid signaling modulates rubber biosynthesis gene expression

This study demonstrated a closely coordinated and complex response of guayule seedlings to salicylic acid involving changes in gene expression. The results demonstrated a concentration-dependent upregulation of cis-prenyltransferase 3 (*CPT3*) and small rubber particle protein (*SRPP*) genes in response to salicylic acid treatment. The pronounced induction of both genes, particularly at 7.5 µM salicylic acid, suggested that salicylic acid might function as an upstream regulator of rubber biosynthesis at the transcriptional level. Previous studies associated elevated endogenous salicylic acid and stress-induced changes in its metabolism with increased rubber transferase activity and enhanced rubber accumulation in guayule, suggesting a regulatory role in rubber biosynthesis [8,13]. *CPT* catalyzes cis-polyisoprene chain elongation, and *SRPP* contributes to rubber particle stabilization and activity [32–34]. In guayule, natural rubber biosynthesis depends on functional CPT-CBP (CPT-binding protein) complexes, with *CPT3* identified as a key candidate in this process [35,36]. In this study, salicylic acid-induced upregulation of *CPT3* and *SRPP* genes, suggested a role in regulating transcription of core rubber biosynthesis genes. Previous studies revealed that *H*. *brasiliensis CPT* genes, particularly *CPT2*, were highly connected *hub* genes within rubber biosynthesis networks and shown strong responsiveness to hormonal and stress-related signals, including ethylene, jasmonates, and salicylic acid [17,37]. Similarly, studies in *Taraxacum kok-saghyz* and *T. brevicorniculatum* have demonstrated that *CPT* and *SRPP* genes responded to defense-related phytohormones, including salicylic acid, and to stress stimuli [38–41]. In addition, the expression patterns of genes involved in rubber biosynthesis in *H*. *brasiliensis* were significantly altered by drought stress [42]. Taken together, these results suggested that hormone-mediated regulation of rubber biosynthesis may be conserved across rubber-producing species [43]. Beyond hormonal signaling, environmental cues, particularly cold, played a critical role in guayule rubber production. Stonebloom and Scheller [11], demonstrated that *AOS*, *CPT*, and *FPS* (*FPPS*) transcripts were significantly up-regulated under simulated winter conditions, while *SRPP* was also significantly induced. This indicated that these four genes formed a coordinated transcriptional module associated with cold-induced rubber production [11]. Salicylic acid is widely recognized as a regulator of plant defense and secondary metabolism, capable of modulating isoprenoid biosynthesis through transcriptional reprogramming [44–46]. The upregulation of *CPT3* and *SRPP* genes observed in this study may have reflected a broader salicylic acid-mediated shift toward secondary metabolite production, where rubber biosynthesis might be enhanced as part of a coordinated stress-adaptive response. However, further studies combining hormone treatments with gene expression analysis and direct measurements of rubber content, latex production, and rubber particle characteristics are needed to confirm the effects of salicylic acid on rubber production.

Farnesyl pyrophosphate synthase (*FPPS*) knockouts and conditional knockdowns revealed buffering within the isoprenoid network, with only moderate reductions in sterols and ubiquinone [47,48], suggesting that *FPPS* functions mainly as a precursor-supplying control node rather than a pathway-specific rate-limiting step. In *H*. *brasiliensis*, *FPPS* expression increased in response to latex tapping, while hormone treatments showed minimal effects on transcript levels [49]. Consistent with these observations, the present study showed moderate changes in *FPPS* expression following salicylic acid treatment, in contrast to the strong, concentration-dependent induction of downstream rubber-specific genes such as *CPT3* and *SRPP*.

Allene oxide synthase (*AOS*) has a distinct regulatory position at the intersection of jasmonate biosynthesis and rubber production [11]. In the present study, *AOS* transcript levels increased in response to salicylic acid in a concentration-dependent manner, with higher salicylic acid levels eliciting stronger transcriptional responses under in vitro conditions. This pattern contrasted with previous reports describing antagonistic effects of salicylic acid on *AOS* expression, particularly under stress conditions or at relatively high hormone concentrations [50–52]. For example, Placido et al., [8] reported that exogenous salicylic acid treatment caused dose-dependent repression of *AOS* expression in *P*. *argentatum*, and RNAi-mediated suppression of *AOS* resulted in reduced rubber particle size and enhanced rubber accumulation. These contrasting outcomes likely reflected differences in salicylic acid concentration, exposure duration, and experimental conditions. It is well known that salicylic acid signaling is concentration-dependent: low-to-moderate salicylic acid levels can promote transcriptional reprogramming without suppressing of jasmonate biosynthesis, whereas higher concentrations intensify antagonistic crosstalk between the salicylic acid and jasmonic acid pathways [53–55]. Consequently, the salicylic acid concentrations used in the present study likely fell within a lower regulatory range, which was expected to modulate gene expression rather than directly suppress AOS enzymatic activity or downstream jasmonate biosynthesis. Recent transcriptomic and network-based studies further supported this interpretation, demonstrating that salicylic acid could reshape jasmonate-associated signaling networks without necessarily promoting or suppressing jasmonic acid production in a linear manner [56,57]. Together with previous observations that *AOS* is up-regulated under simulated winter conditions [11], these results suggested that *AOS* might integrate both hormonal and environmental signals to modulate rubber biosynthesis in guayule. It is noted that the endogenous salicylic acid and jasmonic acid levels were not directly quantified; therefore, confirmation through targeted phytohormone profiling is required.

## Conclusion

Salicylic acid treatment under in vitro conditions altered both vegetative growth and the transcription of genes associated with rubber biosynthesis in guayule. The responses were concentration dependent: lower salicylic acid levels favored plant growth, whereas higher concentrations more strongly induced the expression of key rubber related genes, including *CPT3*, *SRPP*, *FPPS* and *AOS* genes. As this study focused on morphological and transcriptional responses under controlled in vitro conditions, further research is required to determine the broader functional consequences of salicylic acid treatment. Future efforts involving direct measurements of endogenous hormone levels, rubber accumulation, and rubber particle characteristics, along with validation under greenhouse and field conditions, will be essential to elucidate the role of salicylic acid in regulating rubber biosynthesis in guayule. Collectively, these findings suggest that salicylic acid may act as a regulatory signal influencing the expression of key rubber biosynthetic genes, highlighting the potential link between stress related signaling pathways and rubber production.

## Supporting information

S1 File Inclusivity in global research. (DOCX)
